# Control of swine dysentery at national level in Sweden

**DOI:** 10.1186/s13028-024-00769-3

**Published:** 2024-09-05

**Authors:** Per Wallgren

**Affiliations:** 1 Swedish Veterinary Agency, SVA, 751 89 Uppsala, Sweden; 2 Department of Clinical Sciences, Swedish University of Agricultural Sciences, SLU, Box 7054, 750 07 Uppsala, Sweden

**Keywords:** *Brachyspira hyodysenteriae*, Eradication, Pig

## Abstract

**Background:**

Swine dysentery, caused by *Brachyspira hyodysenteriae*, is a severe pig disease. Resistance to tylosins is common and resistance to tiamulin has been reported since the 1990s. Still, dysentery is not notifiable to authorities. The disease therefore escapes control from an overall population perspective. In Sweden, a program that aimed to control dysentery at national level was initiated in 2020, mainly due to the unexpected diagnosis of tiamulin resistant *Brachyspira hyodysenteriae* in 2016.

**Results:**

Through joint efforts of a network including farmers, government, animal health organisations and abattoirs it was concluded that outbreaks of dysentery had taken place in 25 herds between 2016 and 2019. By 1 January 2020, nine of these herds were still not declared free from the disease. From that date, the network decided that *Brachyspira hyodysenteriae* was to be cultured whenever dysentery could be suspected. Thus, 148, 157 and 124 herds were scrutinised for *Brachyspira hyodysenteriae* in 2020, 2021 and 2022, respectively, whereof five, three and two new herds were confirmed positive. By 31 December 2022, four herds were judged as impossible to sanitise. However, they posed no problem since they were identified by the network, pigs to and from these enterprises could be transported without jeopardising other herds. When *Brachyspira hyodysenteriae* was diagnosed in fattening herds purchasing growers, *Brachyspira hyodysenteriae* could not be detected in the delivering herds. That result, together with other observations, indicated that *Brachyspira hyodysenteriae* ought to be regarded as ubiquitous, although at a low level in healthy pigs.

**Conclusions:**

Eradication of dysentery contributed to substantial welfare and financial improvements in affected herds. Dysentery was controlled successfully at national level through the united efforts from competing stake holders, such as different abattoirs and animal health organisations. However, as *Brachyspira hyodysenteriae* was assumed to be ubiquitous, although at a low level in healthy pigs, the duration of the successful control of dysentery was concluded to only be transient. Without permanent monitoring for *Brachyspira hyodysenteriae*, the knowledge of the national status will rapidly decline to the level prior to the initiation of the control program.

## Background

Swine dysentery (SD) is a severe disease that affects the large intestine of pigs [1]. It is caused by *Brachyspira* (*B.*) *hyodysenteriae* and leads to ill-thrift in pigs and to substantial economic losses for farmers [1]. Despite this, SD is not notifiable to authorities and information about the overall incidence of SD in porcine populations is therefore generally lacking. Herds affected by SD may escape detection since competing abattoirs and Animal Health Organisations (AHOs) do not share information with each other. One exception from this is Switzerland, where SD commonly has been eradicated in pig herds, mainly motivated by financial aspects of farmers but also due to external pressure to sanitise [2].

In Sweden, SD was first diagnosed in 1960 [3], but the disease was very rare before 1986. By 1986, Sweden was the first country that banned the use of so-called growth promoters, *i.e.* routine low dose antibiotics in feed with the aim to improve weight gain [4]. Soon thereafter, the treatments of pigs with antibiotics due to post weaning diarrhoea were doubled [5], and subsequently SD became a common diagnosis, especially in weaners and growers [5, 6]. Diagnostics were not sophisticated at that time when the porcine *spirochetes* only included the subspecies *Treponema hyodysenteriae* and *Treponema innocens*.

The effects of SD demanded measures to control the disease in affected herds, and sanitation models that aimed to eradicate SD at herd level were developed already in the late 1980s [5]. The SD sanitations were effective, pigs regained health and *Treponema* spp. were not demonstrated in faecal samples following sanitation. However, controls carried out one year after sanitation generally found *Treponema* spp. in faecal samples collected from apparently healthy pigs (unpublished data) which created pedagogical dilemmas. How to treat apparently healthy herds when *Treponema* spp. was present without causing disease? As a consequence, the annual bacteriological controls of sanitised herds were terminated. Thus, sanitised herds were only monitored for absence of clinical signs of SD by herd veterinarians, and potentially infected herds thereby escaped attention from an overall population perspective.

With time it became evident that differentiation between *Treponema hyodysenteriae* and *Treponema innocens* was more complicated than degree of haemolysis and intensified laboratory methods divided the porcine spirochetes into four major groups [7], whereof group I emanated from pigs with severe diarrhoea and included the reference strain of *B. hyodysenteriae*, and group III was identified in apparently healthy pigs and included the reference strain of *B. innocens*. Groups II and IV were also associated with diarrhoea, whereof group IV was later defined as *B. pilosicoli* [8]. During this process, the initial family name *Treponema* was first changed to *Serpula*, thereafter to *Serpulina* and finally to *Brachyspira*. Onwards, the name *Brachyspira* will be used.

The genome of *B. hyodysenteriae* includes a gene that rapidly develops resistance to tylosins [9]. Tylosin derivates are therefore not suitable for long term use in controlling SD, instead tiamulin derivates have been the drug of choice [10]. However, resistance to tiamulin was reported already in 1996 [11], and tiamulin resistance has been associated to a specific gene of *B. hyodysenteriae* [12]. Thus, herds with severe SD caused by tiamulin-resistant strains of *B. hyodysenteriae* risk to be stamped out due to lack of treatment alternatives [13]. Tiamulin resistance was not observed in Sweden up to 2015 [14], and the general impression was that SD, despite occasional diagnosis of *B. hyodysenteriae*, was rare [15] as well as easy and profitable to control [16]. Therefore, there were no incentives for a national strategy towards SD, with exception for nucleus and multiplier herds merchandising breeding stock to other herds that had been declared free from SD by annual controls by cultivating for presence of *B. hyodysenteriae* since the 1990s.

All of that changed in 2016 when tiamulin-resistant *B. hyodysenteriae* was confirmed in a piglet producing herd with 500 sows [17] that allocated growers to five specialised fattening herds that also became infected [18]. Luckily, the resistant strain was still sensitive to tylosins, and the tiamulin-resistant strain was eradicated without repopulating the sow herd employing high doses of tylvalosin (Aovlosin, ECO Animal Health Europe Ltd, Dublin, Ireland) and intensified biosecurity and hygienic measures [5, 18]. The ability of *B. hyodysenteriae* to rapidly develop resistance to tylosins [9] was confirmed by increasing MIC values to tylosins in isolates of *B. hyodysenteriae* after initiating the program. The fattening herds were sanitised when units were emptied at slaughter [18]. The appearance of tiamulin-resistant *B. hyodysenteriae* provoked an awareness in pig farmers and veterinarians. If this could happen once, it could/would probably occur again and an increasing interest in controlling SD at national level developed. The aim of this study was control SD at national level in Sweden.

## Materials and methods

### Creating a network with the aim to control infections with B. hyodysenteriae

On 30 October 2019, a meeting in Linköping that was organised by Gunnar Johansson (Farm & Animal Health, Linköping, Sweden) and Per Wallgren (Swedish Veterinary Agency (SVA), Uppsala, Sweden) was attended by all stake holders identified as strategic in controlling SD at national level. They included The Swedish Pig Producers Organisation, SVA, all AHOs that worked with pigs and the supply managers of pigs to all larger abattoirs of the country (Table 1). General rules for the strategies of controlling SD were presented and accepted by all stake holders.

**Table 1:** The network that aimed to control dysentery in pigs at national level

*Category*	Organisations		
*Pig farmers*	The Swedish Pig Producers Association		
*Government*	The Swedish Veterinary Agency, SVA		
*Health Organisations*	Farm & Animal Health	Lunden Animal Health	District Veterinarians
*Abattoirs*	Dahlberg Abattoir Ltd	Ginsten Abattoir Ltd	HK Scan
*Abattoirs*	KSL-Ugglarp Ltd	Knårrevången Slaughter Ltd	
*Abattoirs*	Siljan Charcutier Ltd	Skövde Abattoir Ltd	
*Branch company*	The Swedish Meat Producers Enterprises Ltd		

As the competing companies (AHOs and abattoirs) were not interested in sharing data with each other, but also for GDPR reasons, it was decided that SVA should lead and organise the work.

The network was established on 30 October 2019 and was administrated by SVA. All members were informed at every change of the national dysentery status. The network met at least once per year over the internet.

### Optimising the sample strategy with the aim to detect B. hyodysenteriae

The meeting in Linköping decided to optimise the diagnosis of SD. Rectal swabs were to be collected and sent to SVA and analysed for the presence of *B. hyodysenteriae* whenever SD was suspected or could be considered as a differential diagnosis, including whenever signs of blood were seen in faeces of pigs with loose stool. In general, ten samples were analysed per herd. Individual herds could be sampled more than once during a year.

### Diagnostic methods

All analyses regarding *B. hyodysenteriae* were carried out at SVA. In brief, rectal swabs were transported in Aimes medium and inoculated on *Brachyspira* agar plates (blood agar base supplemented with sheep blood, colistin, vancomycin and spectinomycin at 42 ⁰C under anaerobic conditions). Plates were examined after three and six days, spirochaetes with strong haemolysis, indole production and galactosidase activity but without hippurate cleaving capacity and galactosidase activity were defined as *B. hyodysenteriae* [7].

As cultivation of spirochetes is time demanding, PCR wase occasionally used with the aim to speed up the process. The PCR used was based on the tlyA gene of *B. hyodysenteriae* and had a 97% correlation with culture results [19, 20].

### Eradication of SD in herds confirmed with the disease

Sow herds confirmed with SD were sanitised without repopulation employing methods developed in the late 1980s [5]. In brief, it mimicked the sanitation models used to control *Mycoplasma hyopneumoniae* [21–24] complemented with intensified hygienic measures of the furnishing, such as lifting slatted floors when washing and disinfecting units. Growers were sold out and the breeding stock were medicated with tiamulin (Tiamutin, Leo Pharma, Ballerup, Denmark) before being reintroduced to sanitised buildings. Written sanitation protocols were established and communicated to the staff of the affected herds.

Specialised fattening herds with all in/all out production were sanitised by cleaning and disinfection, including lifting slatted floor, as the buildings were emptied following slaughter of batches. If they comprised more than one fattening unit, written biosecurity protocols were established with the aim to avoid contamination of cleansed units.

### Criteria for declaring herds confirmed with SD free from the disease

The meeting in Linköping 2019 decided that herds confirmed with SD should have effectuated three samplings searching for *B. hyodysenteriae* from at least 10 pigs (preferable with diarrhoea if present in herd) with negative results before being declared free from the disease. These demands are still valid for piglet producers and farrow to finish herds.

By 2022 it became evident that the motivation for specialised fattening herds without clinical signs of SD to control for presence of *B. hyodysenteriae* at three consecutive times was low to absent. With the aim to speed up the process of declaring specialised fattening herds free from SD, a new strategy was implemented at an internet meeting with the network held in 2022. Specialised fattening herds could be declared free from SD after one bacterial control of at least 10 pigs (preferable with diarrhoea if present in herd), provided that the herd had been free from clinical signs of SD for six months and that affected units had been washed and sanitised at two different occasions (*i.e.* following two consecutive rearing batches) before the control sampling took place.

### Defining the status of SD in the country by 2019

With the aim to define the incidence of SD in the country, the meeting that took place on 30 October 2019 decided that all AHOs should report outbreaks of SD during the period 2016–2019 to SVA. The information should also include either information on the present status in the confirmed herds or complementing bacterial samplings with the aim to define that status.

All information was compiled into a dataset, that included all herds that had been confirmed with SD during 2016–2019, as well as updated information on the present status of the herds.

### Updating the incidence of SD from 2019

With the aim to update the information on the national status regarding SD, the inventory list of the years 2016–2019 was continuously updated from 1 January 2020. All samples that were analysed with respect to presence of *B. hyodysenteriae* were inserted in the list. These samples emanated from three categories of herds.

1. Annual controls of nucleus and multiplier herds selling breeding stock to other herds with the aim to declare freedom from SD in these herds.
2. Herds with clinical signs that could not exclude SD.
3. Control of herds confirmed with SD, either to confirm the present situation of a herd or with the aim to evaluate the efficacy of measures aimed to eradicate SD.

The results obtained were summarised by SVA. At regular intervals, or whenever transformations of the list of confirmed herds took place, SVA informed the AHO:s and the supply managers of the abattoirs. To streamline that information, also strategic animal transporters were included in the chain of information.

Each time *B. hyodysenteriae* was diagnosed, the herd veterinarian was contacted and the herd was defined as confirmed with SD. Confirmed herds were declared free from the disease once the demands to declare freedom from disease were fulfilled (see above).

### Strategies to minimise spread of SD from affected herds

As mentioned above, all herds diagnosed with SD during 2016–2019, as well as herds later confirmed with the disease were listed. Until declared free from SD, pigs to or from these herds were to be transported at the end of the day and preferably on Fridays. Following transport of pigs from or to herds confirmed with SD, vehicles were to be washed and disinfected before transport of other animals. The aim was to minimise spread of SD from herds affected by the disease.

## Results

### The status of SD in the country by 2019

**Fig. 1:**
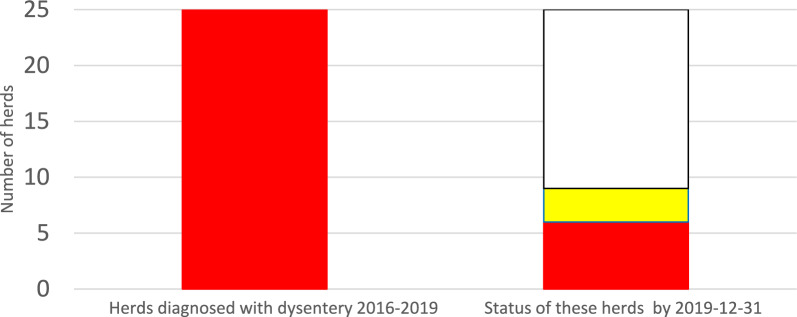
Pig herds in Sweden diagnosed with dysentery (SD) during 2016–2019 and their status by 2019–12-31

During 2016–2019, a total number of 25 herds were confirmed with *B. hyodysenteriae*. Of these, 16 herds had been sanitised and declared free from SD. Another three herds had been sanitised, but were not yet declared free from SD. Yet another six herds were confirmed with SD but had made no attempts to sanitise for the disease. Thus, by 1 January 2020 a total number of nine pig herds were confirmed with SD in Sweden (Fig. 1).

In Fig. 1, red color represents herds diagnosed with dysentery. White represents herds declared free from the disease (n = 16) and yellow represents sanitized herds that not yet had been declared free (n = 3).

### SD in Sweden 2020

**Fig. 2:**
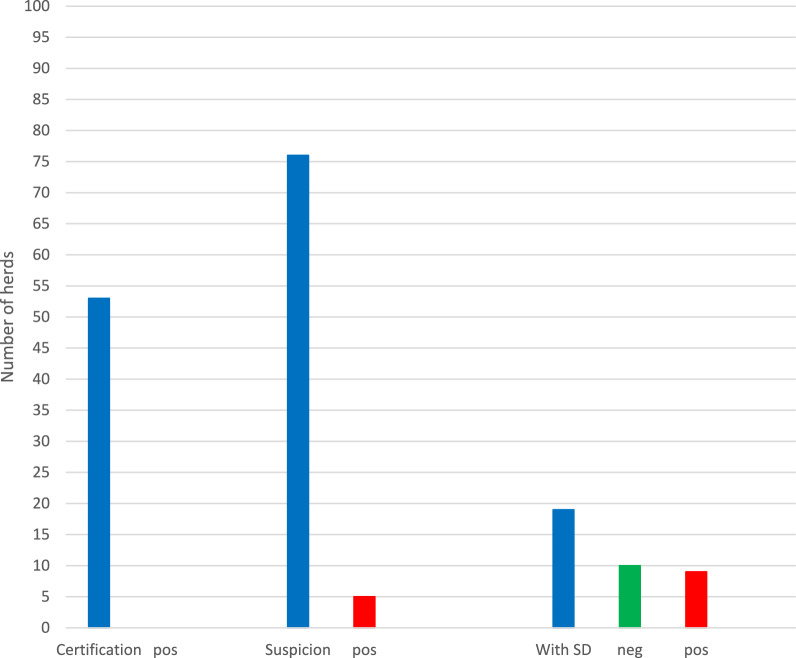
Sampling occasions of herds for dysentery in Sweden during 2020 and their results

During 2020, a total number of 148 herds were analysed for presence of *B. hyodysenteriae* (Fig. 2). *B. hyodysenteriae* was not demonstrated in any of the 53 samplings that represented nucleus or multiplier herds (n = 18) that were screened for SD. In contrast, *B. hyodysenteriae* was demonstrated in five out of 76 samplings carried out in herds previously free from the SD, and in nine out of 19 samplings performed in herds confirmed with SD.

In Fig. 2, blue staples represent all samples collected within category. “Certification” represents monitoring freedom from dysentery in healthy herds selling breeding stock to other herds, “Suspicion” represents herds sampled because of suspicion of dysentery, and “with SD” represents control of herds deemed with dysentery. Red columns represent samples that diagnosed *B. hyodysenteriae* and the green column within the “with SD”-category represents samples where *B. hyodysenteriae* not could be demonstrated.

**Fig. 3:**
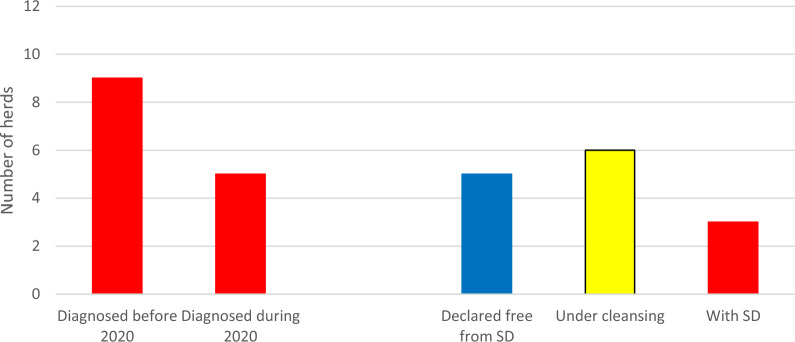
Pig herds in Sweden diagnosed with dysentery (SD) during 2020 and their status by 2020–12-31

In total, five new herds were confirmed with *B. hyodysenteriae* in 2020 (Fig. 2, Table 2). However, another five herds were declared free from the disease. Thereby, the number of herds confirmed with SD by 31 December 2020 remained at nine. Of these, six herds were under cleansing, while three herds lacked strategies for eradicating *B. hyodysenteriae* (Fig. 3).

**Table 2:** Pig herds confirmed with dysentery in Sweden during 2020–2022

*Year*	*Month*	*Herd*	*Comment*
*2020*	February	Fattening herd	Almost subclinical, but blood in faeces of some pigs *B. hyodysenteriae* not found in the delivering herd, but that herd had previously been confirmed with SD
*2020*	March	Integrated herd	Organic herd with outdoor production The herd had previously been suspected of SD
*2020*	March	Integrated herd	40 sows, continuous production. No control of purchases
*2020*	September	Fattening herd	*B. hyodysenteriae* not found in delivering herds
*2020*	December	Piglet producer	*B. hyodysenteriae* not found in the herd delivering gilts
*2021*	January	Integrated herd	Previously confirmed with SD but declared free after three negative samplings in 2020 Again declared free in 2022 after three negative samplings without a total sanitation
*2021*	January	Fattening herd	*B. hyodysenteriae* not found in delivering herds
*2021*	February	Fattening herd	*B. hyodysenteriae* not found in delivering herds
*2022*	January	Fattening herd	*B. hyodysenteriae* not found in delivering herds
*2022*	March	Fattening herd	*B. hyodysenteriae* not found in delivering herds

### SD in Sweden 2021

**Fig. 4:**
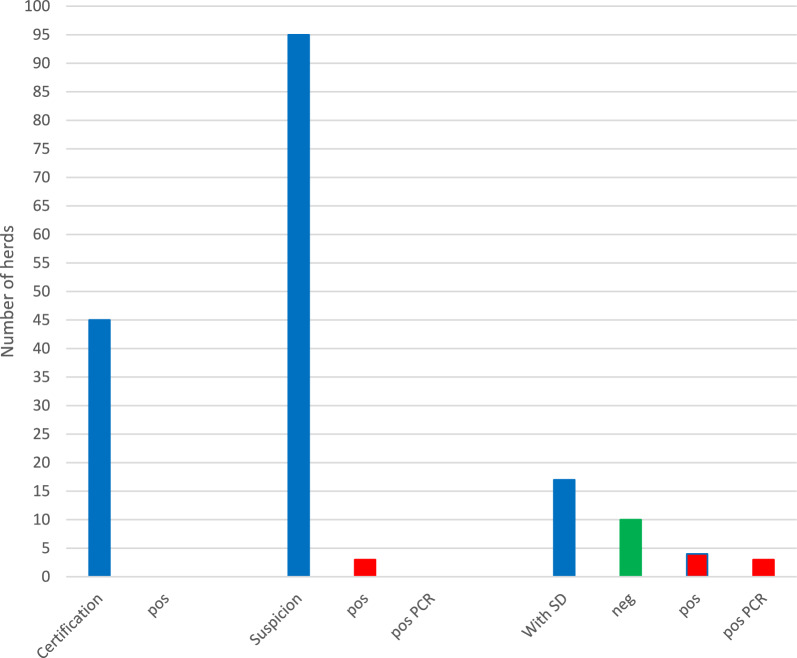
Sampling occasions of herds for dysentery in Sweden during 2021 and their results

During 2021, a total number of 157 herds were analysed for presence of *B. hyodysenteriae* (Fig. 4). *B. hyodysenteriae* was not demonstrated in any of the 45 samplings that represented nucleus or multiplier herds that were screened for SD. Nor was *B. hyodysenteriae* demonstrated in 92 out of 95 samplings representing herds suspected of SD or in 10 out of 17 samplings performed in herds confirmed with SD. In the latter category, *B. hyodysenteriae* was diagnosed by cultivation in four herds and by PCR in three herds.

In Fig. 4, blue staples represent all samples collected within category. “Certification” represents monitoring freedom from dysentery in healthy herds selling breeding stock to other herds, “Suspicion” represents herds sampled because of suspicion of dysentery, and “with SD” represents control of herds deemed with dysentery. Red columns represent samples that diagnosed *B. hyodysenteriae* and the green column within the “with SD”-category represents samples where *B. hyodysenteriae* not could be demonstrated.

In total, *B. hyodysenteriae* was diagnosed in three new herds during 2021 (Fig. 4, Table 2). One of these herds was a previously SD-confirmed integrated herd that had been declared free from *B. hyodysenteriae* following three consecutive samples on 18 June 2020. *B. hyodysenteriae* was again diagnosed in samples collected from diarrhoeic pigs by 21 January 2021. The other two herds were specialised fattening herds that purchased growers at a weight of approximately 30 kg. In both these herds *B. hyodysenteriae* was diagnosed during wintertime (January and February) and shortly after the arrival of growers to previously emptied and cleaned units. The piglet producing herds that had delivered pigs to the affected fattening herds were controlled for presence of *B. hyodysenteriae*, but *B. hyodysenteriae* was not found in these herds.

**Fig. 5:**
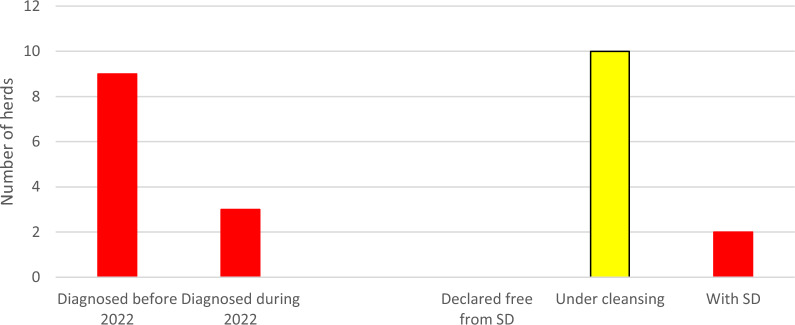
Pig herds in Sweden diagnosed with dysentery (SD) during 2021 and their status by 2021–12-31

No herd was declared free from SD during 2021, but 10 out of the 12 confirmed herds were under cleansing and *B hyodysenteriae* had not been demonstrated in these herds at 10 out of 17 occasions. Sanitation had not been initiated in two herds. Thus, the total number of herds confirmed with SD increased from nine to 12 during 2021 (Fig. 5).

### SD in Sweden 2022

**Fig. 6:**
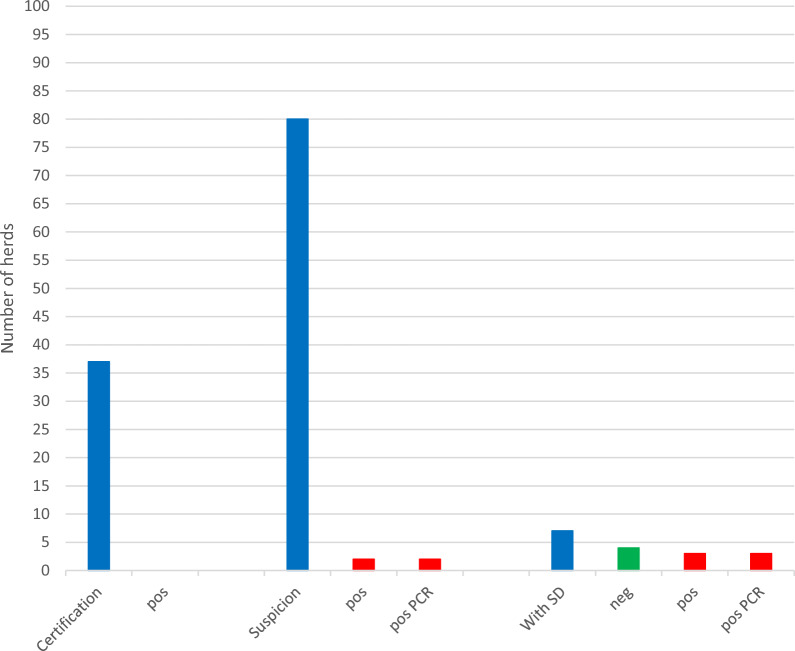
Sampling occasions of herds for dysentery in Sweden during 2022 and their results

During 2022, a total number of 124 herds were analysed for presence of *B. hyodysenteriae* (Fig. 6). *B. hyodysenteriae* was not demonstrated in any of the 37 samplings collected in nucleus or multiplying herds that were screened for SD. Nor was *B. hyodysenteriae* demonstrated in 78 out of 80 samplings representing herds suspected of SD or in four out of seven samplings effectuated in herds confirmed with SD. In the latter category*, B. hyodysenteriae* was demonstrated by both cultivation and by PCR in three herds.

In Fig 6, blue staples represent all samples collected within category. “Certification” represents monitoring freedom from dysentery in healthy herds selling breeding stock to other herds, “Suspicion” represents herds sampled because of suspicion of dysentery, and “with SD” represents control of herds deemed with dysentery. Red columns represent samples that diagnosed *B. hyodysenteriae* and the green column within the “with SD”-category represents samples where *B. hyodysenteriae* not could be demonstrated.

During the winter of 2022 (January and March), *B. hyodysenteriae* was diagnosed, by both cultivation and by PCR, shortly after arrival of growers to previously emptied and cleaned units in two previously healthy specialised fattening herds (Fig. 6, Table 2). The piglet producing herds that had delivered pigs to the affected herds were controlled for presence of *B. hyodysenteriae*, but *B. hyodysenteriae* was not found in these herds.

By 20 January 2022, the integrated herd that was confirmed with SD by 21 January 2021 was again declared free from SD following three consecutive negative samplings. As stated above the herd had previously been declared free from *B. hyodysenteriae* following sanitation and three consecutive negative samplings. The herd was not sanitised following the reappearance of *B. hyodysenteriae* in 2021, but the affected unit was thoroughly cleaned and disinfected when emptied. All negative sampling occasions included samples from the unit where *B. hyodysenteriae* was diagnosed in January 2021.

Following the simplified demands for declaring apparently healthy specialised fattening herds free from SD, six such herds were declared free from *B. hyodysenteriae* during the autumn of 2022.

**Fig. 7:**
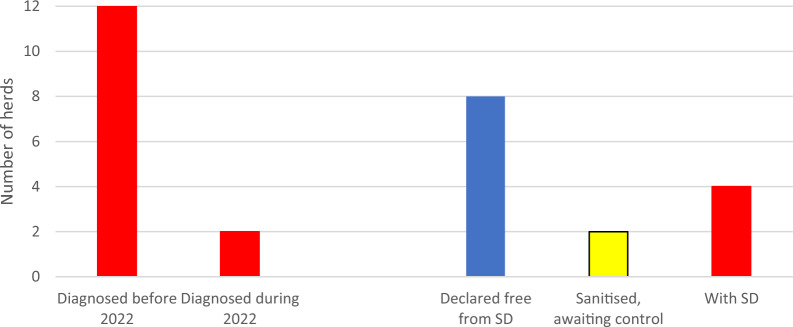
Pig herds in Sweden diagnosed with dysentery during 2022 and their status by 2022–12-31

By the end of 2022, six herds in Sweden were not declared free from SD (Fig. 7). Two of these were the two specialised fattening herds where *B. hyodysenteriae* had been diagnosed in the winter of that year. They were both sanitised and apparently healthy but awaited the results of confirming tests to prove freedom from *B. hyodysenteriae*. The other four herds included:

- Two large, specialised fattening herds judged as impossible to sanitise, as their owners were not interested in sanitation. Both these herds were confirmed with SD before 2020.
- An integrated organic herd rearing pigs outdoors, judged as impossible to sanitise. This herd will close down in 2024.
- A small standalone integrated herd that slaughtered pigs at a small independent abattoir. This constellation was and is isolated from the mainstream of the Swedish pig production.

## Discussion

National strategies to control SD had been discussed earlier but concluded as impossible to implement for commercial reasons (with exception for herds selling breeders to other herds that had been controlled since the 1990s). The control program was in principle initiated because of the diagnosis of tiamulin-resistant *B. hyodysenteriae* in 2016 [17]. When the affected herds had been sanitised [18] and the tiamulin-resistant *B. hyodysenteriae* strain had not been demonstrated for three years despite being searched for [25], the time had come to contact stakeholders within the pig production. The shared fear for tiamulin resistance enabled initiating collective strategies among competing companies and development of mutual strategies aimed to control SD at national level.

Swine dysentery was successfully controlled at national level within a period of three years, which was made possible by the collaboration between different stakeholders with overlapping competencies (Table 1). By 31 December 2022, only six herds were not yet declared free from SD. Of these, two specialised fattening herds were sanitised and awaited a declaration of freedom from the disease. The other four herds were for different reasons judged as impossible to sanitise. However, as they were few and identified by the network that aimed to control SD at national level, they posed no problem. Pigs to and from these enterprises were transported by the end of the day (ideally Fridays) and vehicles used were thoroughly washed and disinfected before transporting other pigs.

Affected sow herds were sanitised without repopulation by breaking the chain of infection between pigs, complemented with intensified hygienic measures of the furnishing, including lifting slatted floors when washing and disinfecting, and reintroduction of tiamulin-treated breeding stock to disinfected facilities [5, 18]. In agreement with other studies [26], attitude and initiative from farmers were crucial for positive results. Written sanitation programs were therefore communicated to all personnel before being initiated. To persuade farmers of the advantages of sanitation, it was also a strength that sanitation of SD had improved the annual income per sow with 251€ in a Swedish farrow to finish herd, which clearly exceeded the sanitation costs of 74€ per sow [16].

However, SD may be difficult to eradicate from a herd without depopulation and thorough cleaning and disinfection before repopulation [27], as also elucidated by the fact that 50 out of 68 SD-sanitations in Switzerland (74%) included a total repopulation [2]. Therefore, the program demanded three consecutive negative samples before declaring diagnosed herds free from SD. This strategy was successfully employed in sow herds. In contrast, specialised fattening herds effectuating all in/all out rearing that sanitised buildings when emptied were found less motivated to effectuate three consecutive samples to certify freedom from SD. As a consequence, the accumulated number of herds confirmed with SD paradoxically increased during 2021 because new cases of SD generally affected specialised fattening herds soon after establishing new batches during winter. Therefore, the demands for declaring specialised fattening herds free from SD was somewhat simplified in 2022. They could be declared free from SD after one bacteriological control, provided that the herd had been free from signs of SD for six months, and that empty units had been washed and sanitised during two consecutive batches before the control sampling took place. This simplification improved the process and, with exception of two large herds with less motivated owners, in principle all specialised fattening herds were declared free from SD during the autumn of 2022.

During 2020–2022, *B. hyodysenteriae* was diagnosed in six previously free specialised fattening herds. Common for these cases were that *B. hyodysenteriae* was diagnosed during the winter, soon after allocation and that *B. hyodysenteriae* was not demonstrated in the piglet producing herds that had sold growers to these herds. Beyond that, *B. hyodysenteriae* was in 2021 only diagnosed in a previously SD-confirmed farrow to finish herd (also during winter) that had been declared free from *B. hyodysenteriae* following three consecutive *B. hyodysenteriae*-negative samplings in 2020. The herd owners showed reluctance to sanitise the whole herd again, but cleaned and disinfected the units where *B. hyodysenteriae* had been diagnosed as they were emptied. Despite not undertaking a total sanitation, the herd was again declared free from *B. hyodysenteriae* following three consecutive negative samplings.

The above demonstrated the complexity of SD. Why was *B. hyodysenteriae* not diagnosed in the piglet producing herds? How could *B. hyodysenteriae* reappear after sanitation and then again disappear in the integrated herd without a total sanitation? Still, these findings were well in line with the fact that *B. hyodysenteriae* can easily be demonstrated in pigs with SD, but generally not in healthy pigs [28] and that it is difficult to induce SD by infecting pigs with *B. hyodysenteriae* solely, while gut content from SD-affected pigs induced the disease in recipients [29]. Accordingly, a synergistic action to other bacteria has been suggested by several authors [29–32]. Modern diagnostic tools such as next generation sequencing (NGS) open up new approaches. NGS has shown that faeces of recently weaned healthy piglets include small but increasing numbers (2.5–5%) of spirochaetes [33, 34], The spirochaetal family include *Brachyspira* spp., and indeed low amounts of *Brachyspira* spp. were demonstrated in healthy piglets by NGS [33, 34]. Bearing these observations in mind, it was tempting to draw a parallel to post weaning diarrhoea (PWD).

Post weaning diarrhoea is a multifactorial disease which is associated with *E. coli* and generally affects pigs around one week post weaning. PWD can be associated with a disturbed intestinal microbiota due to the sudden change from milk to cereals at weaning [35–37]. The coliform microbiota is highly diversified during suckling, but following weaning certain clones will dominate and the diversity of the flora decrease rapidly, which represent a moment of danger as the protection from pathogenic microbes by competitions over nutrients will decrease [38]. The decreased diversity enables us to identify pathogenic strains of *E. coli* as they proliferate at the expense of other strains, and the results obtained in a herd affected by oedema disease were striking. A specific clone of *E. coli* serotype O139 was constantly diagnosed in all piglets affected by oedema disease, while that clone was only demonstrated in one out of 50 apparently healthy piglets of the herd (in a piglet from a litter where oedema disease had been diagnosed [39]). These results indicated that the clone was present in the herd all the time but escaped detection in healthy piglets before weaning due to the high diversity of the intestinal microbiota, as well as in non-affected weaned pigs where other clones dominated the microbiota.

A similar relationship to a disrupted microbiota in spirochaetes would help to understand why we were unable to detect *B. hyodysenteriae* in the piglet producing herds that had delivered growers to the SD affected fattening herds. Indeed, allocation to fattening enterprises represents a moment of chronic stress that affects pigs negatively and also decrease the capacity of their immune system [40]. It could also help to understand the unexpected reoccurrence of clinical SD occasionally seen when combatting the disease, including the reappearance of *B. hyodysenteriae* in the integrated herd that was re-confirmed with SD in 2021 and from which the disease and presence of *B. hyodysenteriae* appeared to disappear without sanitation.

This view could also help to understand the sudden onset of clinical SD in Sweden following the ban of feed additives in 1986 [4]. The rapid onset of clinical SD all over the country following the ban of growth promoters [5, 6] indicated that *B. hyodysenteriae* had been present in many herds already before the ban, but also suggested that the bacteria had been controlled using low dose antibiotics in feed which had been used prior to 1986. With time, the design of feed to weaners, in general attained a similar control of *B. hyodysenteriae* [41]. This was mainly achieved by decreasing the protein levels [41]. Additionally, feed that limits the amounts of fermentable ø entering the large intestine has been shown to reduce clinical SD [42, 43].

Such reasoning could also explain why defined pathogenic strains of *B. hyodysenteriae* occasionally have been isolated in apparently healthy herds [44], and it would also explain why diets based on cooked rice and animal protein has been proven effective in preventing SD [45, 46]. In contrast, soya-bean based diets have been shown to predispose for diarrhoea [47–49].

Despite that SD is a severe disease, the reasoning above indicated that *B. hyodysenteriae* is included in the normal microbiota of pigs, although rare in apparently healthy pigs. This could be regarded as a change of the paradigm, but the possibilities that *B. hyodysenteriae* could be harboured in the intestine without causing disease has repeatedly been suggested [15, 44, 50] and that view facilitated the understanding of the disease from an epidemiological view. However, it also created new challenges. How to declare freedom from an infection that is ubiquitous? The insight that developed with time was that we might have to accept that *B. hyodysenteriae* is to be regarded as ubiquitous, but also that we do not want to experience the consequences of clinical SD.

Thus, the project aimed to a reach a condition when *B. hyodysenteriae* could not be demonstrated in the faeces of pigs and thereby effectively prevent spread of clinical SD within and between herds. As evident from the results obtained in this study, we observed no inconsistent results that could create pedagogical dilemmas as previously experienced in the 1980s (unpublished data). Indeed, unspecific diagnostic, but also methods with increased sensitivity, may create interpretation problems since every finding of *B. hyodysenteriae* must be carefully attended. As cultivating *B. hyodysenteriae* is time consuming, PCR-methods have been used with the aim to speed up diagnostics. During 2021, *B. hyodysenteriae* was occasionally demonstrated by PCR but not by cultivation, but also vice versa, in samples from herds confirmed with SD. As the correlation between cultivation and the PCR used had been reported to be 97% [19], PCR was not assumed to cause diverging results from cultivating, which was confirmed as the results obtained by cultivation and PCR were consistent in 2022. Time will tell if increased sensitivity of molecular methods will create interpretation problems in the future control of SD.

## Conclusion

In conclusion, dysentery was successfully controlled at national level in Sweden. This success required the united efforts of farmers, government and competing abattoirs and AHOs. Apart from controlling SD, the work also contributed to substantial welfare and financial improvements of affected herds.

However, the duration of the successful control of SD at national level is only transient, and the knowledge on the national status of SD will rapidly decline to the level prior to the initiation of this project if the monitoring for SD will be terminated. As *B. hyodysenteriae* was concluded to be ubiquitous, although at a low level in healthy pigs, long-lasting knowledge of the SD status of a population demands continuous monitoring for *B. hyodysenteriae*. Consequently, this project has taken on an administrative phase that maintains the monitoring strategies carried out during 2020–2022.

## Data Availability

Not applicable.

## Abbreviations

AHOs: Animal Health Organisations
GDPR: General Data Protection Regulation
NGS: Next generation sequencing
PCR: Polymerase chain reaction
PWD: Post-weaning diarrhoea
SD: Swine dysentery
spp.: Species
SVA: Swedish Veterinary Agency
